# Distribution of α_2_-Adrenergic Receptors in the Living Human Brain Using [^11^C]yohimbine PET

**DOI:** 10.3390/biom13050843

**Published:** 2023-05-15

**Authors:** Chloé Laurencin, Sophie Lancelot, Inès Merida, Nicolas Costes, Jérôme Redouté, Didier Le Bars, Philippe Boulinguez, Bénédicte Ballanger

**Affiliations:** 1 Université de Lyon, 69622 Lyon, France; chloe.laurencin@chu-lyon.fr (C.L.); sophie.lancelot@univ-lyon1.fr (S.L.); philippe.boulinguez@univ-lyon1.fr (P.B.); 2 Université Claude Bernard Lyon 1, 69100 Villeurbanne, France; 3 INSERM U1028, Lyon Neuroscience Research Center (CRNL), 69000 Lyon, France; 4 CNRS UMR5292, Lyon Neuroscience Research Center (CRNL), 69000 Lyon, France; 5 Hôpital Neurologique Pierre Wertheimer, Service de Neurologie C, Centre Expert Parkinson, Hospices Civils de Lyon, 69677 Bron, France; 6 CERMEP-Imagerie du Vivant, 69500 Bron, France; ines.merida@cermep.fr (I.M.); costes@cermep.fr (N.C.); redoute@cermep.fr (J.R.);; 7 Hospices Civils de Lyon, 69677 Bron, France

**Keywords:** α_2_-adrenoceptor, [^11^C]yohimbine, hybrid PET/MRI, human, cartography, in vivo

## Abstract

The neurofunctional basis of the noradrenergic (NA) system and its associated disorders is still very incomplete because in vivo imaging tools in humans have been missing up to now. Here, for the first time, we use [^11^C]yohimbine in a large sample of subjects (46 healthy volunteers, 23 females, 23 males; aged 20–50) to perform direct quantification of regional alpha 2 adrenergic receptors’ (α_2_-ARs) availability in the living human brain. The global map shows the highest [^11^C]yohimbine binding in the hippocampus, the occipital lobe, the cingulate gyrus, and the frontal lobe. Moderate binding was found in the parietal lobe, thalamus, parahippocampus, insula, and temporal lobe. Low levels of binding were found in the basal ganglia, the amygdala, the cerebellum, and the raphe nucleus. Parcellation of the brain into anatomical subregions revealed important variations in [^11^C]yohimbine binding within most structures. Strong heterogeneity was found in the occipital lobe, the frontal lobe, and the basal ganglia, with substantial gender effects. Mapping the distribution of α_2_-ARs in the living human brain may prove useful not only for understanding the role of the NA system in many brain functions, but also for understanding neurodegenerative diseases in which altered NA transmission with specific loss of α_2_-ARs is suspected.

## 1. Introduction

The noradrenergic (NA) system has been implicated in the regulation of brain regions involved in numerous motor and non-motor functions, in particular arousal, sensory processing, attention, working memory, cognitive control, reward, mood, and emotion [1,2,3,4]. Accordingly, disruptions of NA functions have been associated with several disorders, with a potentially important role in neurodegenerative diseases [5,6,7,8,9,10]. This makes the NA system an important molecular target for drug development [11,12,13,14,15]. However, knowledge of the neurofunctional basis of the NA system on which such an endeavor is based is still incomplete and highly controversial. A major reason for the lack of knowledge is the lack of specific in vivo imaging tools in humans.

Detailed mapping of anatomical distribution of α_2_-adrenergic receptors (ARs) in the human brain is essential for understanding the neurochemistry of neurodegenerative and neuropsychiatric disorders. Until now, it has only been studied ex vivo using quantitative autoradiography [16,17,18,19,20,21,22,23,24]. Although these studies have provided important insights, they have substantial limitations inherent to the technique. Findings obtained with autoradiography have highlighted dramatic species differences in the levels of α_2_-ARs [18] in several brain areas, making it difficult to extrapolate findings from animals to humans and complicating the interpretation of behavioral studies in rodents. The results obtained with autoradiography in humans are based on fragmentary analyses, and caveats are found in the restrictions of the tissue samples analyzed, such as samples of the human frontal cortex only related to Brodmann’s area 10 [22] or Brodmann’s area 9 [17,25]. Finally, autoradiography findings are limited by the sample size that can be used (ranging from 2 [26] to 22 [21] subjects).

In vivo imaging of the NA system has recently become feasible in humans with the development of a novel PET radiotracer: the [^11^C]yohimbine [27,28]. [^11^C]yohimbine binds with high selectivity to all α_2_-ARs subtypes [29]. This represents a great opportunity to fill in missing data in the living human brain by directly quantifying regional α_2_-AR availability. Here, for the first time, we use [^11^C]yohimbine in a large sample of subjects to map the distribution of α_2_-ARs in the living human brain.

## 2. Materials and Methods

### 2.1. Participants

Forty-six healthy subjects (23 males (mean age ± SD, 35.1 ± 9.4; range 20–50 y) and 23 females (mean age ± SD, 35.5 ± 9; range 20–50 y)) were selected to participate in the study. Male and female subjects were recruited to achieve a homogeneous age distribution between 20 and 50 years old. Exclusion criteria were as follows: ferromagnetic implanted materials, claustrophobia, pregnancy, history of noradrenergic medications, history of dependence on alcohol or other drugs of abuse, diagnosis of other neurological or psychiatric disorders, and history of head trauma. None of the participants were smokers.

### 2.2. PET Procedures

[^11^C]yohimbine was synthesized as previously described [29]. The radiochemical purities of syntheses used for the study were greater than 95%, with corresponding molar activities of 70 ± 29 GBq/μmol at the end of synthesis. All subjects received an intravenous bolus injection of 370 MBq ± 10% of [^11^C]yohimbine. List-mode PET data were acquired, during the 90 min from the injection of the tracer, simultaneously with 3T MRI data (Dixon T1, anatomic MPRAGE T1) on a Siemens mMR Biograph system.

### 2.3. Image Processing

Raw PET data were motion corrected [30], and then rebinned into 24 time frame (variable length frames, 8 × 15 s, 3 × 60 s, 5 × 120 s, 1 × 300 s, 7 × 600 s) sinograms for dynamic reconstruction. Images were reconstructed using 3D ordinary Poisson-ordered subset expectation maximization (OP-OSEM 3D), incorporating the system point spread function using 3 iterations of 21 subsets. Sinograms were corrected for scatter, randoms, normalization, and attenuation [31]. Reconstructions were performed with a zoom of 2 in a matrix of 172 × 172 voxels, yielding a voxel size of 2.03 × 2.03 × 2.08 mm^3^, with a 4 mm 3D post-reconstruction Gaussian filtering. Individual MRI T1 was normalized to the MNI space (Montreal Neurological Institute template of the International Consortium for Brain Mapping Project) using the Segment function of SPM 12 [32]. Labeling of the structural brain regions was performed using the multi-atlas propagation with enhanced registration (MAPER) methodology [33], and the 83-region Hammers atlas [34]. After segmentation, regional time–activity curves (TAC) were extracted, and the non-displaceable binding potential (BP_ND_) obtained with the Simplified Reference Tissue Model (SRTM) was calculated in the different ROIs with the corpus callosum taken as the reference region [28].

### 2.4. Statistical Analysis

Statistical analysis was performed using Rstudio (https://github.com/rstudio/rstudio, accessed on 1 September 2022). Results with *p* < 0.05 were considered statistically significant. First, we conducted a coarse-grained analysis by applying a 13 ROI (Hippocampus, Occipital Lobe, Cingulate Gyrus, Frontal Lobe, Parietal Lobe, Thalamus, Parahippocampus, Insula, Temporal Lobe, Basal Ganglia, Amygdala, Cerebellum, Raphe nucleus) × 2 side (left, right) × 2 sex (male, female) ANOVA. Then, we performed fine-grained analyses by fractionating the 6 main ROIs according to the Hammers atlas [34]. For each one of these main ROIs (Occipital lobe, Cingulate gyrus, Frontal Lobe, Parietal Lobe, Temporal Lobe, Basal Ganglia), a *n* Subregion (varying from 2 to 9 according to the ROI) × 2 side (left, right) × 2 gender (male, female) ANOVA was performed.

For illustrative purposes only, SPM12 was used for whole brain voxel-based analysis on the spatially normalized and smoothed parametric BP images of the 46 control subjects. To assess sex-related variability, we applied a two-sample *t* test between males and females, with individual BP values taken as covariates for interindividual adjustment. Statistical parametric maps of the *t* statistic were computed with a threshold of *p* = 0.005 uncorrected at the voxel level.

## 3. Results

### 3.1. Regional ROI Analysis

Table 1 shows BP_ND_ values for the 13 anatomical regions. The results show a main effect of region (F(11, 4090) = 118.53, *p* < 0.001) (Table 2) as well as a main effect of sex (F(1, 4090) = 29.11, *p* < 0.001) without significant difference between sides (*p* = 0.1). An interaction between sex and region (F(11, 4090) = 2.63, *p* < 0.005) indicates that significant differences between females and males are observed in the cerebellum (*p* = 0.01), the frontal and parietal lobes (*p* < 0.001), and the hippocampus (*p* < 0.001). As there was no significant difference between left and right BP_ND_, values were pooled to determine the mean BP_ND_ and the standard deviation (SD) within each ROI (Table 1).

### 3.2. Subregional ROI Analyses

Within the occipital lobe, a significant main effect of region is observed (F(2, 264) = 87.5; *p* < 0.001) showing that [^11^C]yohimbine BP_ND_ is not different between the cuneus (0.69 ± 0.17) and the lingual gyrus (0.66 ± 0.17), two regions characterized by a stronger BP than the lateral parts of the occipital lobe (0.40 ± 0.14, ps < 0.001).

Within the cingulate gyrus, a significant main effect of region is reported (F(1, 176) = 24.6; *p* < 0.001), showing that [^11^C]yohimbine BP_ND_ is higher in its posterior part (PCC, 0.61 ± 0.14) than in its anterior part (ACC, 0.51 ± 0.13).

Within the frontal lobe, significant main effects of sex and regions are observed, without interaction. Males have overall higher [^11^C]yohimbine BP_ND_ than females (F(1, 1068) = 16.7 *p* < 0.001). Among the frontal subregions, the highest [^11^C]yohimbine BP_ND_ is found within the straight gyrus (0.65 ± 0.15), followed by the orbital gyrus (0.58 ± 0.16), the presubgenual ACC (0.57 ± 0.16), and the middle frontal gyrus (0.54 ± 0.15). Intermediate [^11^C]yohimbine BP_ND_ is observed in the superior frontal gyrus (0.51 ± 0.15), the inferior frontal gyrus (0.49 ± 0.13), the precentral gyrus (0.43 ± 0.14), and the subgenual ACC (0.42 ± 0.13), while the lowest [^11^C]yohimbine BP_ND_ is observed in the subcallosal area (0.36 ± 0.13). *p*-value thresholds are shown in Table 3.

Within the parietal lobe, only a significant effect of sex is observed (F(1, 352) = 14.16; *p* < 0.001), with males (0.51 ± 0.14) having overall higher [^11^C]yohimbine BP_ND_ than females (0.45 ± 0.13).

Within the temporal lobe, significant main effects of sex (F(1, 624) = 4.2; *p* = 0.04), side (F(1, 624) = 15.03; *p* < 0.001), and region (F(4, 624) = 22.2; *p* < 0.001) are observed with no significant interaction between the three independent variables. Males (0.39 ± 0.15) have overall higher [^11^C]yohimbine BP_ND_ than females (0.36 ± 0.14). BP_ND_ is higher on the right side (0.40 ± 0.14) than on the left side (0.36 ± 0.14). Finally, [^11^C]yohimbine BP_ND_ is higher in the posterior temporal lobe (0.47 ± 0.13) than in a group of subregions composed of the fusiform gyrus (0.41 ± 0.13), the superior temporal gyrus (0.38 ± 0.15), and in the middle and inferior temporal gyrus (0.37 ± 0.12) which show intermediate binding levels. A lower [^11^C]yohimbine BP_ND_ was reported in the anterior temporal lobe (0.32 ± 0.13).

Within the basal ganglia, a significant main effect of region is reported (F(4, 440) = 60.54; *p* < 0.001) showing that [^11^C]yohimbine BP_ND_ is higher in the nucleus accumbens (0.41 ± 0.14), the putamen (0.40 ± 0.17), and the pallidum (0.39 ± 0.15) than in the substantia nigra (0.19 ± 0.14) and the caudate (0.17 ± 0.12).

We summarized all the BP_ND_ values in Table 4.

### 3.3. Whole Brain Illustrations

**Figure 1:**
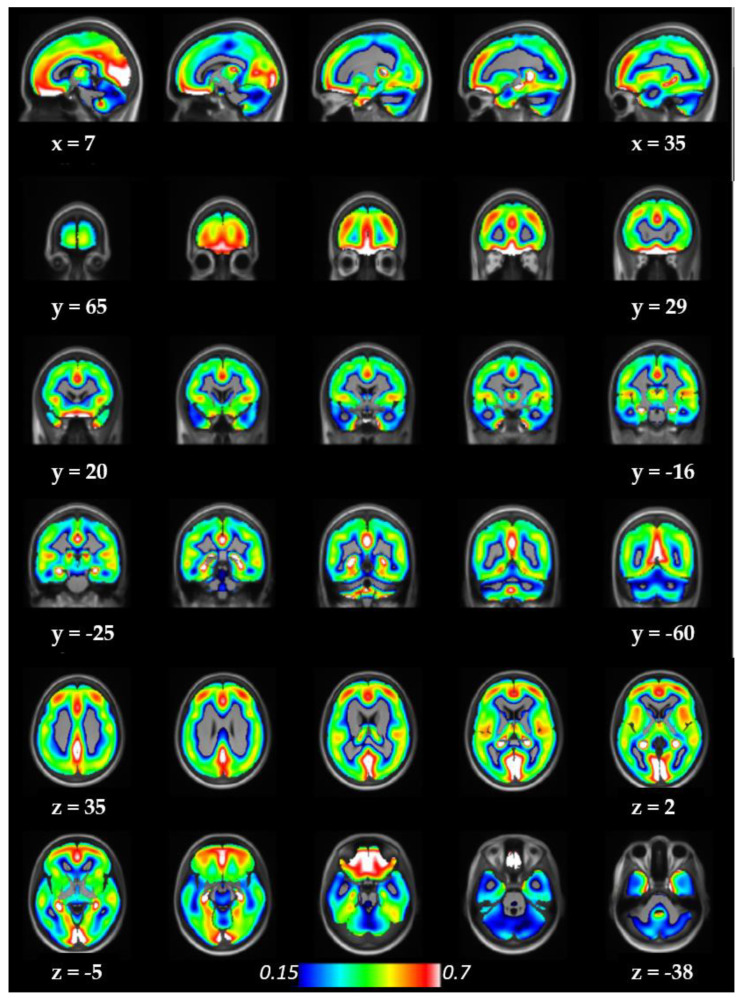
Widespread distribution of α_2_-ARs in the living human brain. Color bar gives estimates of BP_ND_ in units of mL.cm^−3^. In order to facilitate the use of this map, the average parametric image of BP_ND_ values of this dataset can be downloaded and used for any purpose with a simple request to the corresponding author.

The distribution of α_2_-ARs in the living human brain can be illustrated using the average parametric images of [^11^C]yohimbine BP_ND_ values overlaid on an average T1-weighted image of the 46 subjects (Figure 1).

**Figure 2:**
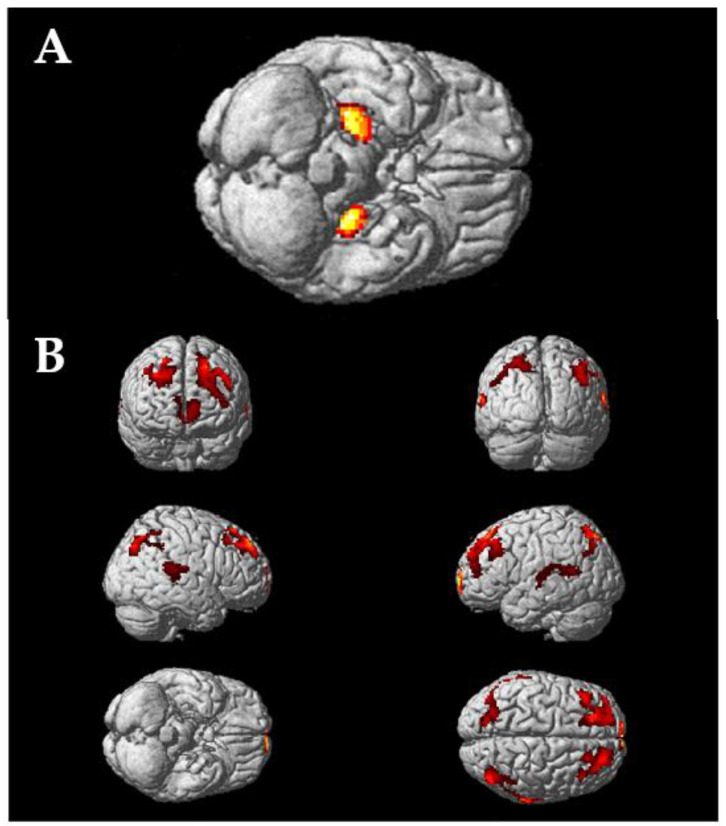
Statistical parametric maps comparing females to males. Data are surface-rendered onto the T1-weighted average reference brain provided by SPM12 using a voxel threshold of *p* < 0.005 (uncorrected) without cluster correction (k = 100 voxels). (**A**) Increase in [^11^C]yohimbine binding in female compared with male subjects is observed bilaterally in the hippocampus. (**B**) Increase in [^11^C]yohimbine binding in male compared with female subjects is observed in the orbital gyrus, superior frontal gyrus, and the middle and superior temporal gyrus, as well as the superior parietal and angular gyrus.

The gender effect found in the main coarse-grained and fine-grained statistical analyses can be illustrated using the statistical parametric maps comparing females to males (Figure 2).

## 4. Discussion

In this paper, we present for the first time a complete human brain mapping of the α_2_-ARs in vivo. Our data confirm the widespread distribution of these receptors throughout the human brain. The regional distribution of [^11^C]yohimbine binding is broadly consistent with the major findings of post-mortem human brain studies [17,19,20,21,22], further validating the use of the radiotracer. More specifically, the present dataset provides a complete and refined picture of subregional variations in α_2_-ARs based on 35 anatomical subdivisions of the Hammers’ probabilistic atlas [34,35]. This statistical mapping is intended to provide a tool for interpreting current issues on the physiological bases of NA functions in the human brain and for conducting future investigations of the role of α_2_-ARs in regulating NA neurotransmission in healthy and clinical conditions. Furthermore, this statistical mapping also raises new issues such as gender effects.

### 4.1. Regional Distribution of [^11^C]yohimbine

Important regional variations of α_2_-ARs availability are observed. The highest [^11^C]yohimbine binding was seen in the hippocampus followed by the occipital lobe, the cingulate gyrus, and the frontal lobe. Regions with moderate levels of specific binding included the parietal lobe, the thalamus, the parahippocampus, the insula, and the temporal lobe. Low [^11^C]yohimbine binding was found in the basal ganglia, amygdala, cerebellum, and raphe nucleus. This broad overview does not contradict quantitative autoradiographic studies [16,18,20,21,24].

### 4.2. Subregional Variations in α_2_-ARs Availability

With the exception of the parietal lobe showing remarkable homogeneity (with intermediate to high levels of binding), parcellating the brain into anatomical subregions pinpoints important variations of [^11^C]yohimbine binding within each structure.

The occipital lobe presents a substantial variation of specific binding ranging from very high to intermediate/low, with the medial structures of the visual cortex (cuneus and lingual gyrus) showing the highest binding of all ROIs, whereas the lateral parts of the occipital lobe are characterized by a binding level comparable to structures of the basal ganglia. This strong heterogeneity has been ignored in previous studies, either because only medial regions were examined (e.g., primary visual cortex) [20,21,23] or because global indexes of occipital binding were calculated [24].

The frontal lobe is also characterized by high heterogeneity (Table 3 and Figure 2). A high level of binding was found in the presubgenual ACC and in the straight, orbital, and middle frontal gyri. A medium level of binding was found in the subgenual ACC and in the inferior, superior, and prefrontal gyri. A low level of binding was found in the subcallosal area. These variations were not detected, or even taken in account, in previous autoradiographic studies of the frontal cortex with only a few numbers of subregions of interest (e.g., BA 9 in [17,36,37]; BA 8–9 in [38]; BA 10 in [22]). This observation may prove crucial for understanding the complex and controversial roles of the NA system in the many functions supported by the frontal lobe [39,40,41,42,43].

Interestingly, while the basal ganglia as a whole shows [^11^C]yohimbine binding lying in the low range (Table 1), it is worth mentioning that there are important regional variations between the different nuclei. While the nucleus accumbens, the putamen and pallidum show intermediate binding, and the caudate and the substantia nigra exhibit almost negligible binding (Table 4). This complements the former observation obtained with experimental animal studies, which revealed higher levels of the α_2A_-ARs in the nucleus accumbens than in the caudate [44].

Finally, subregions of the thalamus could not be assessed with the Hammers atlas. However, in contrast to the above data, heterogeneous distribution of α_2_-ARs is expected from human [21] and animal literature [45,46], with concentrations in the midline, pulvinar, and posterior (intralaminar) nuclei as well as in visual and auditory relay nuclei (geniculate nuclei).

### 4.3. New Issues

Our consistent sample of subjects allowed for statistical comparisons between women and men. For the first, differences were found in the hippocampus, cerebellum, and frontal and parietal lobes. Interestingly, while the [^11^C]yohimbine binding in the hippocampus was higher for females than males, the opposite pattern was observed in the other regions.

The higher BP_ND_ could reflect changes in both pre- and postsynaptic receptors. On the one hand, it can be interpreted as reflecting a higher density of α_2_-ARs, but on the other hand, higher BP_ND_ values can also be interpreted as reflecting a higher availability of α_2_-ARs sites due to a lower concentration of NA (given that [^11^C]yohimbine is sensitive to endogenous variations of NA concentration [47]). This effect could be explained by the fact that central α_2_-ARs function would be attenuated by estrogen concentration and, as a consequence, would be relatively “blunted” in females. Some arguments come from animal studies showing that hypothalamic α_2_-ARs decrease following estrogen treatment in ovariectomized rats [48,49], leading α_2_-AR-mediated inhibition of NA release [50,51]. Further arguments come from clinical studies showing a gender effect in the prevalence of α_2_-AR-related disorders, such as mood and anxiety [52], which are almost twice as high in females than in males [53]. Our results highlight the need to consider this gender effect in all types of studies of the NA system.

### 4.4. Limitations

The main limitation of the present study, and by extension of the use of [^11^C]yohimbine PET, is the fact that it does not differentiate between the different subtypes of α_2_-ARs that have been characterized pharmacologically (α_2A_, α_2B_, α_2C_) [54]. The physiological relevance of these subtypes cannot be fully elucidated without specific radioligands, but the differences that have already been identified between the different α_2_ receptor subtypes must be acknowledged for interpreting [^11^C]yohimbine PET data. In particular, the α_2A_-subytpe is known to be the main inhibitory presynaptic feedback receptor [55,56,57] with α_2C_ to a less extent. These two subtypes are also known as heteroceptors that inhibit the release of dopamine and serotonin [58,59] making the problem even more complex. In addition, there are α_2_-ARs both at the pre- and postsynaptic sites, which always makes interpretation of the results difficult. Nevertheless, it is broadly considered that the presynaptic inhibitory autoreceptors located on noradrenergic neurons in the locus coeruleus play a crucial role in the regulation of the release and synthesis of noradrenaline, while the postsynaptic α_2_-ARs located in the widely distributed projection areas of the neurons throughout the cortex modulate the signaling pathways [60,61]. Finally, it must be acknowledged that we did not apply partial volume correction, meaning that these data must be interpreted with caution, in particular with regard to the relative low binding in small structures or to the absence of differences between nearby regions due to the spillover effect.

## 5. Conclusions

The α_2_-ARs play a key role in regulating NA neurotransmission [62,63], but their in vivo imaging in humans was not possible until now. Providing a technical solution, [^11^C]yohimbine PET allowed for the mapping of their distribution in the living human brain, which will help interpreting past and future investigations on the role of the NA system in numerous brain functions in healthy as well as in various clinical conditions. This is all the more important as altered NA transmission with specific loss of α_2_-ARs is currently suspected to play a critical role in both the symptoms and progression of some neurodegenerative and mood disorders [9,22].

## Figures and Tables

**Table 1:** Regional distribution of α_2_-ARs in the human brain.

	[^11^C]yohimbine BP_ND_		
	Females	Males	All
Hippocampus	**0.67 ± 0.23**	**0.55 ± 0.17**	0.61 ± 0.21
Occipital Lobe	0.57 ± 0.21	0.60 ± 0.20	0.58 ± 0.20
Cingulate Gyrus	0.55 ± 0.14	0.56 ± 0.15	0.56 ± 0.14
Frontal Lobe	**0.50 ± 0.17**	**0.54 ± 0.16**	0.52 ± 0.17
Parietal Lobe	**0.45 ± 0.13**	**0.51 ± 0.14**	0.48 ± 0.14
Thalamus	0.46 ± 0.16	0.49 ± 0.13	0.48 ± 0.15
Parahippocampus	0.42 ± 0.12	0.44 ± 0.12	0.43 ± 0.12
Insula	0.42 ± 0.13	0.43 ± 0.15	0.42 ± 0.14
Temporal Lobe	0.36 ± 0.14	0.39 ± 0.15	0.38 ± 0.14
Basal Ganglia	0.30 ± 0.18	0.32 ± 0.18	0.31 ± 0.18
Amygdala	0.29 ± 0.10	0.30 ± 0.10	0.30 ± 0.10
Cerebellum	**0.23 ± 0.18**	**0.31 ± 0.12**	0.27 ± 0.16
Raphe	0.22 ± 0.16	0.26 ± 0.18	0.24 ± 0.17

Bold values indicate an effect of sex.

**Table 2:** Statistical differences in [^11^C]yohimbine binding between regions.

	Hippocampus	Occipital Lobe	Cingulate Gyrus	Frontal Lobe	Parietal Lobe	Thalamus	Parahippocampus	Insula	Temporal Lobe	Basal Ganglia	Amygdala	Cerebellum	Raphe
Hippocampus													
Occipital Lobe	ns												
Cingulate Gyrus	ns	ns											
Frontal Lobe	***	***	ns										
Parietal Lobe	***	***	***	***									
Thalamus	***	***	*	ns	ns								
Parahippocampus	***	***	***	***	ns	ns							
Insula	***	***	***	***	***	ns	ns						
Temporal Lobe	***	***	***	***	***	***	ns	***					
Basal Ganglia	***	***	***	***	***	***	***	***	***				
Amygdala	***	***	***	***	***	***	***	***	**	ns			
Cerebellum	***	***	***	***	***	***	***	***	***	ns	ns		
Raphe	***	***	***	***	***	***	***	***	***	**	ns	ns	

ns: nonsignificant (ps > 0.1), *** = *p* < 0.0005, ** = *p* < 0.005, * = *p* < 0.05.

**Table 3:** Differences in [^11^C]yohimbine binding between subregions of the frontal lobe.

	Inferior Frontal Gyrus	Middle Frontal Gyrus	Orbital Gyrus	Precentral Gyrus	Presubgenual ACC	Straight Gyrus	Subcallosal Area	Subgenual ACC	Superior Frontal Gyrus
Inferior Frontal Gyrus									
Middle Frontal Gyrus	ns								
Orbital Gyrus	****	ns							
Precentral Gyrus	ns	****	****						
Presubgenual ACC	*	ns	ns	****					
Straight Gyrus	****	****	***	****	**				
Subcallosal Area	****	****	****	*	****	****			
Subgenual ACC	ns	****	****	ns	****	****	ns		
Superior Frontal Gyrus	ns	ns	**	*	ns	****	****	***	

(ns: nonsignificant, **** = *p* < 0.001, *** = *p* < 0.005, ** = *p* < 0.01, * = *p* < 0.05).

**Table 4:** Subregional distribution of α_2_-ARs in the human brain in fractionated ROIs. Bold values indicate an effect of sex.

	[^11^C]yohimbine BP_ND_		
	Females	Males	All
Cuneus	0.67 ± 0.17	0.71 ± 0.16	0.68 ± 0.17
Lingual Gyrus	0.65 ± 0.18	0.67 ± 0.16	0.66 ± 0.17
Straight Gyrus	0.65 ± 0.15	0.65 ± 0.14	0.65 ± 0.14
Posterior Cingulate Cortex	0.60 ± 0.15	0.62 ± 0.14	0.61 ± 0.14
Hippocampus	**0.67 ± 0.23**	**0.55 ± 0.17**	0.61 ± 0.21
Orbital Gyrus	**0.55 ± 0.17**	**0.60 ± 0.14**	0.58 ± 0.16
Presubgenual ACC	0.56 ± 0.18	0.57 ± 0.15	0.57 ± 0.16
Middle Frontal Gyrus	**0.49 ± 0.14**	**0.58 ± 0.16**	0.54 ± 0.15
Anterior Cingulate Cortex	0.51 ± 0.12	0.51 ± 0.14	0.51 ± 0.13
Superior Frontal Gyrus	**0.49 ± 0.15**	**0.53 ± 0.15**	0.51 ± 0.15
Superior Parietal Gyrus	**0.47 ± 0.13**	**0.52 ± 0.14**	0.50 ± 0.14
Inferior Frontal Gyrus	**0.46 ± 0.11**	**0.51 ± 0.14**	0.49 ± 0.13
Supramarginal Gyrus	**0.46 ± 0.13**	**0.50 ± 0.14**	0.48 ± 0.14
Angular Gyrus	**0.44 ± 0.14**	**0.51 ± 0.15**	0.48 ± 0.15
Thalamus	0.46 ± 0.16	0.49 ± 0.13	0.48 ± 0.15
Posterior Temporal Lobe	**0.46 ± 0.13**	**0.48 ± 0.13**	0.47 ± 0.13
Postcentral Gyrus	**0.43 ± 0.13**	**0.49 ± 0.14**	0.46 ± 0.14
Parahippocampus	0.42 ± 0.12	0.44 ± 0.12	0.43 ± 0.12
Precentral Gyrus	**0.40 ± 0.13**	**0.46 ± 0.14**	0.43 ± 0.14
Subgenual ACC	0.42 ± 0.13	0.41 ± 0.12	0.42 ± 0.13
Insula	0.42 ± 0.13	0.43 ± 0.15	0.42 ± 0.14
Fusiform gyrus	0.41 ± 0.12	0.41 ± 014	0.41 ± 0.13
Nucleus Accumbens	0.40 ± 0.16	0.41 ± 0.13	0.41 ± 0.14
Lateral Occipital Lobe	0.39 ± 0.13	0.42 ± 0.14	0.40 ± 0.14
Putamen	0.37 ± 0.18	0.43 ± 0.17	0.40 ± 0.17
Pallidum	0.37 ± 0.14	0.41 ± 0.16	0.39 ± 0.15
Superior Temporal Gyrus	**0.36 ± 0.14**	**0.39 ± 0.16**	0.38 ± 0.15
Middle and Inferior Temporal Gyrus	0.37 ± 0.12	0.36 ± 0.13	0.37 ± 0.12
Subcallosal Area	0.36 ± 0.14	0.35 ± 0.12	0.35 ± 0.13
Anterior Temporal Lobe	**0.30 ± 0.13**	**0.33 ± 0.13**	0.32 ± 0.13
Amygdala	0.29 ± 0.10	0.30 ± 0.10	0.30 ± 0.10
Cerebellum	**0.23 ± 0.18**	**0.31 ± 0.12**	0.27 ± 0.16
Raphe	0.22 ± 0.16	0.26 ± 0.18	0.24 ± 0.17
Substantia Nigra	0.20 ± 0.14	0.19 ± 0.14	0.19 ± 0.14
Caudate Nucleus	0.15 ± 0.14	0.19 ± 0.09	0.17 ± 0.12

## Data Availability

The authors will be happy to support request for a formal data sharing agreement.
